# Neural oscillations as a signature of efficient coding in the presence of synaptic delays

**DOI:** 10.7554/eLife.13824

**Published:** 2016-07-07

**Authors:** Matthew Chalk, Boris Gutkin, Sophie Denève

**Affiliations:** 1 Institute of Science and Technology Austria, Klosterneuburg, Austria; 2 École Normale Supérieure, Paris, France; 3 Center for Cognition and Decision Making, National Research University Higher School of Economics, Moscow, Russia; University College London, United Kingdom

**Keywords:** neural oscillations, neural coding, computational neuroscience, None

## Abstract

Cortical networks exhibit 'global oscillations', in which neural spike times are entrained to an underlying oscillatory rhythm, but where individual neurons fire irregularly, on only a fraction of cycles. While the network dynamics underlying global oscillations have been well characterised, their function is debated. Here, we show that such global oscillations are a direct consequence of optimal efficient coding in spiking networks with synaptic delays and noise. To avoid firing unnecessary spikes, neurons need to share information about the network state. Ideally, membrane potentials should be strongly correlated and reflect a 'prediction error' while the spikes themselves are uncorrelated and occur rarely. We show that the most efficient representation is when: (i) spike times are entrained to a global Gamma rhythm (implying a consistent representation of the error); but (ii) few neurons fire on each cycle (implying high efficiency), while (iii) excitation and inhibition are tightly balanced. This suggests that cortical networks exhibiting such dynamics are tuned to achieve a maximally efficient population code.

**DOI:** http://dx.doi.org/10.7554/eLife.13824.001

## Introduction

Oscillations are a prominent feature of cortical activity. In sensory areas, one typically observes 'global oscillations' in the gamma-band range (30–80 Hz), alongside single neuron responses that are irregular and sparse (Buzsáki and Wang, 2012; Yu and Ferster, 2010). The magnitude and frequency of gamma-band oscillations are modulated by changes to the sensory environment (e.g. visual stimulus contrast (Henrie and Shapley, 2005) and behavioural state (e.g. attention [Fries et al., 2001]) of the animal. This has led a number of authors to propose that neural oscillations play a fundamental role in cortical computation (Fries, 2009; Engel et al., 2001). Others argue that oscillations emerge as a consequence of interactions between populations of inhibitory and excitatory neurons, and do not perform a direct functional role in themselves (Ray and Maunsell, 2010).

A prevalent theory of sensory processing, the 'efficient coding hypothesis', posits that the role of early sensory processing is to communicate information about the environment using a minimal number of spikes (Barlow, 1961). This implies that the responses of individual neurons should be as asynchronous as possible, so that they do not communicate redundant information (Simoncelli and Olshausen, 2001). Thus, oscillations are generally seen as a bad thing for efficient rate coding, as they tend to synchronise neural responses, and thus, introduce redundancy.

Here we propose that global oscillations are a necessary consequence of efficient rate coding in recurrent neural networks with synaptic delays.

**Figure 1.**
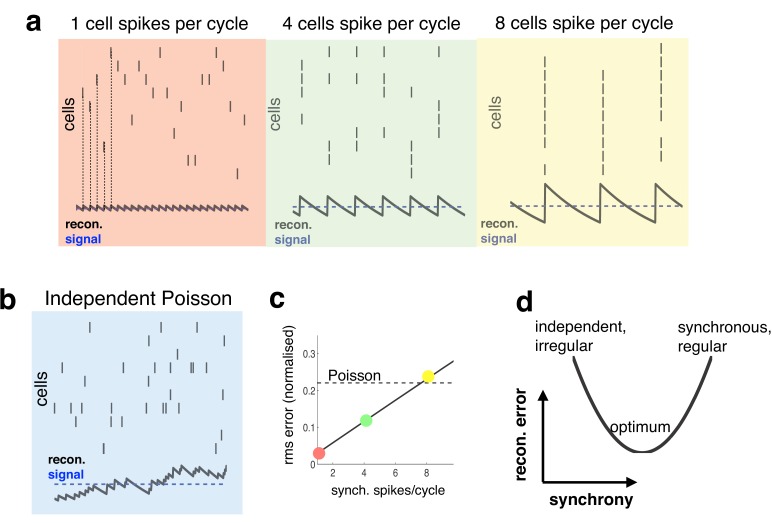
Relationship between synchrony and coding accuracy. (**a**) Each panel illustrates the response of 10 neurons. An encoded sensory variable is denoted by a horizontal blue line. Each spike fired by the network increases the sensory reconstruction by a fixed amount, before it decays. Greatest accuracy is achieved when the population fires at regular intervals, but no two neurons fire together (left). Coding accuracy is reduced when multiple neurons fire together (middle and right panels). (**b**) Same as panel a, but where neurons show independent poisson variability. (**c**) Reconstruction error (root-mean squared error divided by the mean) for a regular spiking network shown in panel a, versus the number of synchronous spikes on each cycle. A horizontal line denotes the performance when neurons fire with independent Poisson variability. (**d**) Cartoon illustrating tradeoff between trade-off faced by neural networks. **DOI:** http://dx.doi.org/10.7554/eLife.13824.002

In general, to avoid communicating redundant information, neurons driven by common inputs should actively decorrelate their spike trains. To illustrate this, consider a simple set-up in which neurons encode a common sensory variable through their firing rates, with a constant value added to the sensory reconstruction each time a neuron fires a spike (Figure 1a).

With a constant input, the reconstruction error is minimized when the population fires spikes at regular intervals, while no two neurons fire spikes at the same time (as in Figure 1a, left panel, in red). To achieve this ideal, however, requires incredibly fast inhibitory connections, so that each time a neuron fires a spike it suppresses all other neurons from firing (Boerlin et al., 2013). In reality, inhibitory connections are subject to unavoidable delays (e.g. synaptic and transmission delays), and thus, cannot always prevent neurons from firing together. Worse, in the presence of delays, inhibitory signals, intended to prevent neurons from firing together, can actually have the reverse effect of *synchronising* the network, so that many neurons fire together on each cycle (as in Figure 1a, middle and right panels). This is analogous to the so-called ‘hipster effect’ where a group of individuals strive to look different from each other, but due to delayed reaction times, end up making similar decisions and all looking alike (Touboul and Effect, 2014).

Spiking synchronicity generally has a negative effect on coding performance. For example, Figure 1c shows how, in the regular spiking network described above, coding error increases with the number of synchronous spikes per cycle (while firing rate is held constant). It is thus tempting to conclude that neural networks should do everything possible to avoid synchronous firing. However, one also observes that a completely asynchronous network, in which neurons fire with independent Poisson variability (Figure 1b), performs far worse than the regular spiking network, even when multiple neurons fire together on each cycle (Figure 1c, horizontal dashed line).

Thus, to perform efficiently, neural networks face a trade-off (Figure 1d). On the one hand, recurrent connections should coordinate the activity of different neurons in the network, so as to achieve an efficient and accurate population code. On the other hand, in the presence of synaptic delays, it is important that these recurrent signals do not overly synchronize the network, as this will reduce coding performance.

Here, we show that, in a network of leaky integrate-and-fire neurons (LIF) optimized for efficient coding, this trade-off is best met by adding noise to the network (e.g. via random fluctuations in membrane potential, or synaptic failure) to ensure that: (i) neural spike trains are entrained to a global oscillatory rhythm (for a consistent representation of global information), but (ii) only a small fraction of cells fire on each oscillation cycle. In this regime, individual neurons fire irregularly (Shadlen and Newsome, 1998) and exhibit weak pairwise correlations (Schneidman et al., 2006), despite the presence of rhythmic population activity. Moreover, excitation and inhibition are tightly balanced on each oscillation cycle, with inhibition lagging excitation by a few milliseconds (Atallah and Scanziani, 2009; Wehr and Zador, 2003). Thus, ‘global oscillations’ come about as a direct consequence of efficient rate coding, in a recurrent network with synaptic delays.

## Results

### Efficient coding in an idealized recurrent network

It is instructive to first consider the behaviour of an idealized network, with instantaneous synapses. For this, we consider a model proposed by Boerlin et al., in which a network of integrate-and-fire neurons is optimized to efficiently encode a time varying input. As the model has already been described elsewhere (Boerlin et al., 2013) we restrict ourselves to outlining the basic principles, with a mathematical description reserved for the **Materials and methods**.

**Figure 2.**
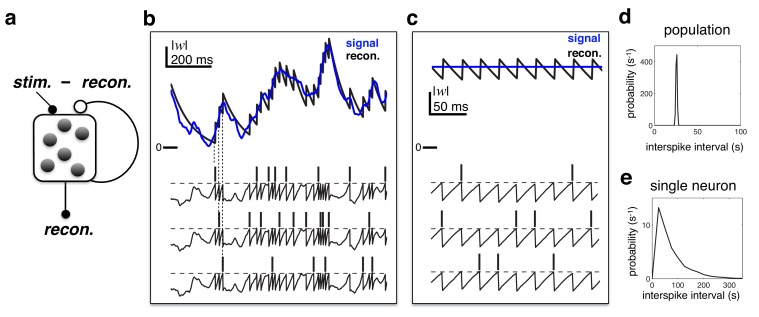
Efficient coding in a recurrent neural network. (**a**) Schematic of network. Inhibitory recurrent connections are represented by an open circle. Excitatory feed-forward connections are represented by closed circles. (**b**) Stimulus (blue) and neural reconstruction (black) on a single trial. The spikes and membrane potential for each cell are shown in separate rows. Vertical dashed lines illustrate how each spike alters the neural reconstruction. (**c**) Same as (**b**), but with a constant input (also note the change of temporal scale). (**d**–**e**) Distribution of inter-spike intervals in population (**d**) and single-cell (**e**) spike trains. **DOI:** http://dx.doi.org/10.7554/eLife.13824.003

Underlying the model is the idea that downstream neurons should be able to reconstruct the input to the network by performing a linear summation of its output spike trains. To do this efficiently (i.e. with as few spikes as possible), the spiking output of the network is fed-back and subtracted from its original input (Figure 2a). In consequence, the total input to each neuron is equal to a ‘prediction error’; the difference between the original input and the network reconstruction. This prediction error is also reflected in neural membrane potentials. When a neuron’s membrane potential becomes larger than a constant threshold, then it fires a spike; recurrent feedback then reduces the prediction error encoded by other neurons, preventing them from firing further redundant spikes.

To illustrate the principles underlying the model, we consider a network of three identical neurons. Figure 2b shows how spikes fired by the network are ‘read-out’, to obtain a continuous reconstruction. Each time a neuron fires a spike, it increases the reconstruction by a fixed amount, decreasing the difference between the input and the neural reconstruction. Immediately after, feedback connections alter the membrane potentials of other neurons, to ensure that they maintain a consistent representation of the error, and do not fire further spikes (Figure 2b, lower panel). As a result, membrane potentials are highly correlated, while spikes are asynchronous.

Figure 2c shows the behaviour of the network in response to a constant input. To optimally encode a constant input, the network generates a regular train of spikes (as in the left panel of Figure 1a), resulting in a narrow distribution of population inter-spike intervals (ISIs) (Figure 2d). Neural membrane potentials, which encode a common prediction error, fluctuate in synchrony, with a frequency dictated by the population firing rate (Figure 2c, lower panel). However, as only one neuron fires per cycle, the spike trains of *individual neurons* are irregular and sparse, resulting in a near-exponential distribution of single-cell ISIs (Figure 2e).

### Efficient coding with synaptic delays

In real neural networks, recurrent inhibition is not instantaneous, but subject to synaptic and transmission delays. Far from being a biological detail, even very short synaptic delays can profoundly change the behaviour of the idealized efficient coding network, and pose fundamental limits on its performance.

**Figure 3.**
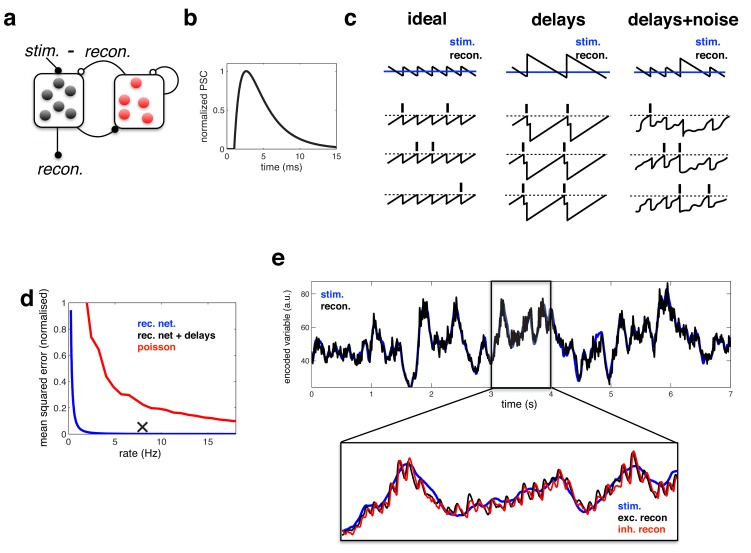
Coding performance of an excitatory/inhibitory network with synaptic delays. (**a**) Schematic of network connectivity. Excitatory neurons and inhibitory neurons are shown in black and red, respectively. Connections between different neural populations are schematised by lines terminating with solid (excitatory connections) and open (inhibitory connections) circles. (**b**) The postsynaptic current (PSC) waveform used in our simulations. (**c**) Schematic, illustrating how delays affect the decoding performance of the network. (**d**) Mean-squared reconstruction error (normalized by variance of encoded signal) versus mean firing rate for an ‘ideal’ efficient coding network with instantaneous synapses (blue), an efficient coding network with finite synaptic delays (black cross) independent Poisson units whose firing rate varies as a fixed function of the feed-forward input (red). (**f**) Stimulus and neural reconstruction for the recurrent network, with synaptic delays. (**inset**) Excitatory (black) and inhibitory (red) neural reconstructions, during a short segment from this trial. **DOI:** http://dx.doi.org/10.7554/eLife.13824.004

To render the idealized model biologically feasible, we extended it in two ways. First, to comply with Dale’s law, we introduced a population of inhibitory neurons, which mediates recurrent inhibition (Figure 3a). In our implementation, excitatory and inhibitory populations both encode two separate reconstructions of the target variable, an ‘excitatory reconstruction’ and an ‘inhibitory reconstruction’. Inhibitory neurons, which receive input from excitatory neurons, fire spikes in order to predict and cancel the inputs to the excitatory population. Consequently, the inhibitory reconstruction closely tracks fluctuations in the excitatory reconstruction (see **Materials and methods**).

Second, and more importantly, we replaced the instantaneous synapses in the idealized model with continuous synaptic dynamics, as shown in Figure 3b (see **Materials and methods**). As stated previously, adding synaptic delays substantially alters the performance of the network. Without delays, recurrent inhibition prevents all but one cell from firing per oscillation cycle, resulting in an optimally efficient code (Figure 3c, left panel). With delays however, inhibition cannot always act fast enough to prevent neurons firing together. As a result, neural activity quickly becomes synchronised, and the sensory reconstruction is destroyed by large population-wide oscillations (Figure 3c, middle panel). To improve performance, one can increase the membrane potential noise and spike threshold, so as to reduce the chance of neurons firing together (Figure 3c, right panel). Too much noise, however, and the firing of different neurons becomes uncoordinated, and network performance is diminished (see later).

We compared the performance of the efficient coding network (with excitatory/inhibitory populations and synaptic delays) to: (i) an ‘ideal’ model with no delays and (ii) a ‘rate model’, consisting of a population of independent Poisson units whose firing rate varies as a function of the feed-forward input (see **Materials and methods**).

In the Poisson network, random fluctuations in firing rate degrade the reconstruction, resulting in large coding error. Increasing the population firing rate increases the signal-to-noise ratio, and thus, also decreases the reconstruction error (Figure 3d, red). In contrast, in the ‘ideal’ efficient coding network, noise fluctuations are automatically corrected for by the recurrent connections (see Materials and methods). Thus, in this network, the only source of inaccuracy comes from the discreteness of the code (where each spike adds a fixed quantity to the readout), leading to a small error (Figure 3d, blue). Finally, while synaptic delays increase the coding error (relative to the ideal efficient coding network), the error remains significantly smaller than for a Poisson network with the matching rate (Figure 3d, black cross).

Figure 3e illustrates the ability of the efficient coding network to track a time varying input signal. Zooming in to a 1 s period within the trial (lower inset), one observes rhythmic fluctuations in the excitatory and inhibitory neural reconstructions. These fluctuations are essentially the same phenomena as observed for the ideal network, where the neural reconstruction fluctuated periodically around the target signal following the arrival of each new spike (Figure 2c). However, with synaptic delays, several neurons fire together before the arrival of recurrent inhibition. As a result, oscillations are slower and larger in magnitude than for the idealised network, where only one neuron fires on each cycle.

### Oscillations and efficient coding

We sought to quantify the effect of oscillations on coding performance. To do this, we varied parameters of the model, so as to alter the degree of network synchrony, while keeping firing rates the same. In the main text we illustrate the effect of adding white noise to the membrane potentials (while simultaneously varying the spike threshold, to maintain constant firing rate; see **Materials and methods**).

**Figure 4.**
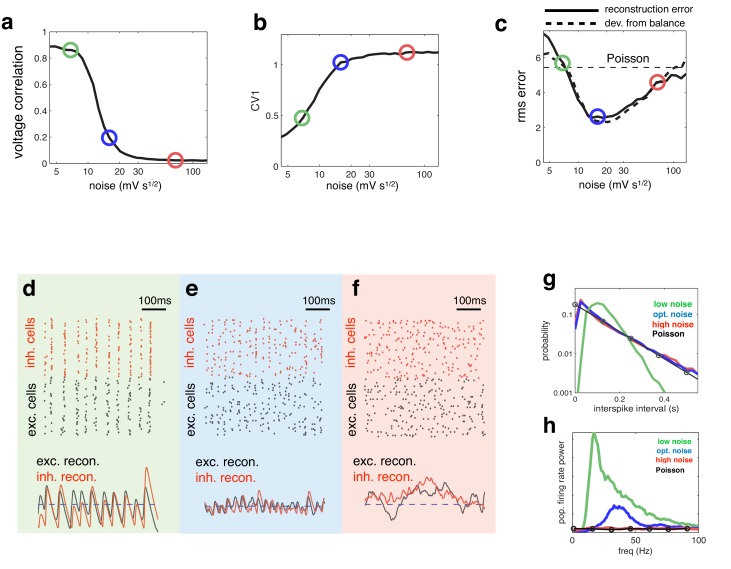
Effect of varying noise amplitude on network dynamics and coding performance. (**a**) Pairwise voltage correlations, versus injected noise amplitude. (**b**) Coefficient of variation in inter-spike intervals, versus injected noise amplitude. (**c**) Root-mean-square (rms) reconstruction error (solid line), plotted alongside rms difference between excitatory and inhibitory reconstructions (dashed line), versus injected noise amplitude. Horizontal dashed line shows the rms reconstruction error for a population of Poisson units with matched firing rates. (**e**–**f**) (upper panels) Spiking response of inhibitory (red) and excitatory (black) neurons with low (**d**), medium (**e**), and high (**f**) noise amplitude (indicated by open circles in panels **a**–**c**). (lower panels). Inhibitory (red) and excitatory (black) neural reconstructions, alongside target stimulus (blue dashed line). (**g**) Distribution of single-cell inter-spike intervals in each noise condition. The prediction for a population of Poisson units is shown in black. Note the log-scale. (**h**) Power spectrum of population firing rate, in each noise condition. The power spectrum for a population of Poisson units is shown in black. **DOI:** http://dx.doi.org/10.7554/eLife.13824.005

Increasing the magnitude of the membrane potential noise desynchronized the network activity, resulting in a reduction in pairwise voltage correlations (Figure 4a). With increased noise, single neural spike trains also became more irregular, reflected by an increase in the spiking CV (Figure 4b).

Coding performance, however, varied non-monotonically with the noise amplitude. The reconstruction error followed a u-shaped curve, being minimised for a certain intermediate level of noise (Figure 4c, solid curve). For this intermediate noise level, coding performance was significantly better than a network of independent Poisson units with a matching firing rate (horizontal dashed line). Interestingly, deviations between excitation and inhibition followed a similar u-shape curve, being minimised for the same intermediate noise level (Figure 4c, thick dashed curve). Thus, optimal coding was achieved when the balance between excitatory and inhibition was the tightest. Further, at the optimal level of noise, the spiking CV value was near unity (Figure 4b), implying irregular (near-poisson) single cell responses.

To further understand the effect of varying noise amplitude, we plotted the network responses and neural reconstruction in three regimes: with low, intermediate, and high noise (indicated by green, blue and red circles respectively in Figure 4a–c).

With low noise, neural membrane potentials were highly correlated, leading many neurons to fire together on each oscillation cycle (Figure 4d, upper panel). As a result, the neural reconstruction exhibited large periodic fluctuations about the encoded input, leading to poor coding performance (Figure 4d, lower panel).

At the other extreme, when the injected noise was very high, the spike trains of different neurons were uncorrelated (Figure 4f, upper panel). As, in this regime, effectively no information was shared between neurons, inhibitory and excitatory reconstructions were decoupled, and coding performance was similar to a population of independent Poisson units (Figure 4f, lower panel).

In the intermediate noise regime, for which performance was optimal, spikes were aligned to rhythmic fluctuations in the prediction error, but few neurons fired on each cycle (Figure 4e, upper panel). These dynamics were reflected by a near-exponential distribution of interspike-intervals (Figure 4e), coupled with a narrow peak in the population firing rate spectrum (Figure 4e). In this regime, rhythmic fluctuations in the neural reconstruction were small in magnitude, and there was a tight coupling between inhibitory and excitatory reconstructions (Figure 4e, lower panel).

### Manipulating synaptic failures and neural noise

**Figure 5.**
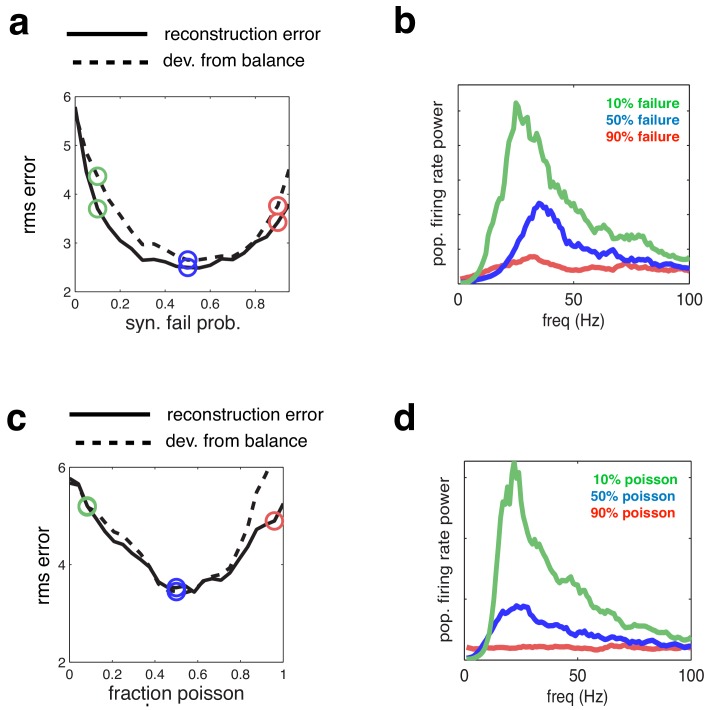
Effect of varying network synchrony in different ways. (**a**–**b**) Effect of varying the probability of synaptic failure. Panel **a** shows the root-mean-square (rms) reconstruction error (solid line), plotted alongside the rms difference between excitatory and inhibitory reconstructions (dashed line). Panel **b** shows the firing rate spectrum with varying probability of synaptic failure (indicated by open circles in panel **a**). (**c**–**d**) Same as panels **a**–**b**, but where we varied the fraction of cells that fired randomly (i.e. with Poisson spike trains). **DOI:** http://dx.doi.org/10.7554/eLife.13824.006

To assess the generality of the results shown in Figure 4, we manipulated the degree of synchrony in the network in different ways. First, we varied the synaptic reliability, by varying the probability that a presynaptic spike led to a change in the postsynaptic membrane potential (Figure 5a–b). Note that the average recurrent input received by each neuron varies proportionally with the synaptic failure probability. Thus, to correct for this, and retain balance in the network, the magnitude of recurrent connection was scaled inversely with the synaptic failure probability (see Materials and methods).

Next, we selected a subset of cells to fire with random, Poisson distributed, firing rates (and matching rate) (Figure 5c–d). For both manipulations, the added membrane potential noise was held constant (equal to the ‘low-noise’ condition indicated by green open circle in Figure 4a–c).

We observed qualitatively similar changes in coding performance and network synchrony, regardless of how we manipulate noise in the network. In both cases, the coding error was smaller at an intermediate level of noise, for which there was tightest balance between inhibition and excitation (Figure 5a and c).

### Oscillatory neural dynamics

**Figure 6.**
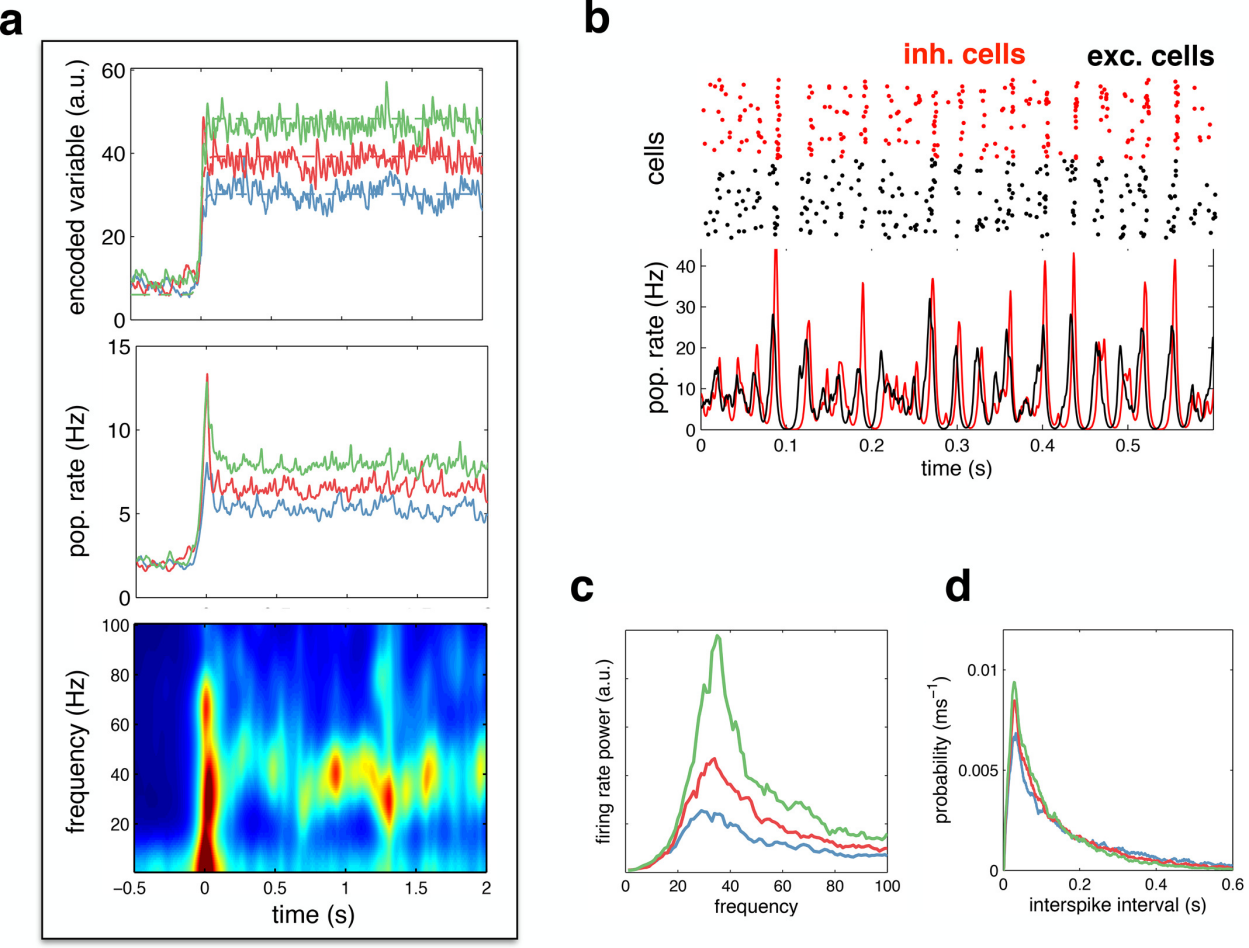
Spiking response to a constant input. (**a**) (**top**) Neural reconstruction (solid lines) of a presented constant stimulus (dashed lines), before and after stimulus onset. Low, medium and high amplitude stimuli are shown in blue, red and green, respectively. (**middle**) Population firing rate, for each stimulus amplitude. (**bottom**) Spectrogram of population firing rate. (**b**) The upper panel shows a raster-gram of excitatory (black) and inhibitory (red) responses, during a 0.6 s period of sustained activity. The lower panel shows the instantaneous firing rates of the excitatory and inhibitory populations. (**c**) Power spectrum of excitatory population firing rate during the sustained activity period, for each stimulus amplitude. (**d**) Average distribution of single-cell inter-spike intervals, for each stimulus amplitude. **DOI:** http://dx.doi.org/10.7554/eLife.13824.007

We investigated the behaviour of the network in the optimal noise regime, shown in Figure 4e. Figure 6a shows the network reconstruction and population firing rates in response to a low (blue), medium (red) and high (green) amplitude stimulus, with the optimal noise amplitude (i.e. 17 mV $s^{1/2}$). The population firing rate was characterised by a transient peak following stimulus onset, followed by decay to a constant value. A spectrogram of the population firing rate (Figure 6a, lower panel) reveals the presence of 30–50 Hz oscillations during the period of sustained activity. Figure 6b plots the spiking response and population firing rates during a 600 ms period of sustained activity. Here, one clearly sees correlated rhythmic fluctuations in excitatory (black) and inhibitory (red) activity. The strength of these oscillations increases with stimulus amplitude (Figure 6c). Nevertheless, for all input amplitudes, individual neurons fired irregularly, with a near-exponential distribution of inter-spike intervals (Figure 6d).

**Figure 7.**
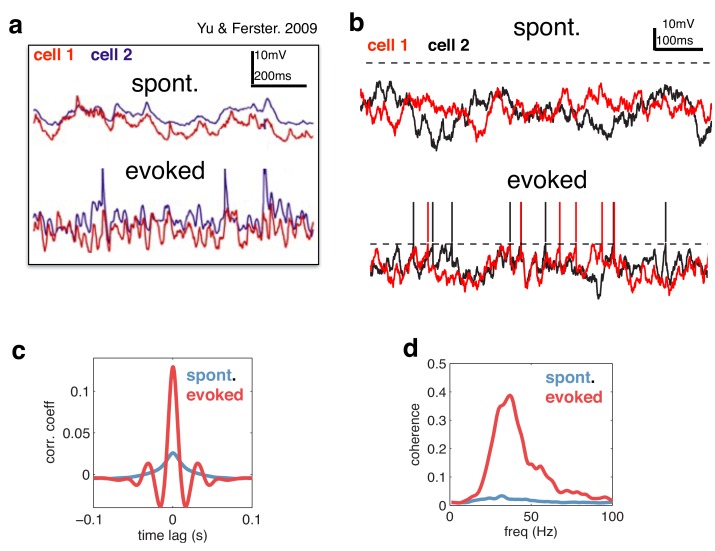
Voltage responses in response to a constant input. (**a**) Data from Yu & Ferster (Neuron, 2010), showing voltage traces of two V1 cells, in the absence (top), and presence (bottom) of a visual stimulus. (**b**) Voltage traces of two cells in the model in absence (top) and presence (bottom) of a feed-forward input. (**c**) Average pairwise cross-correlation between membrane potentials, in spontaneous and evoked condition. (**d**) Average coherence spectrum between pairs of voltage traces, in spontaneous and evoked condition. **DOI:** http://dx.doi.org/10.7554/eLife.13824.008

We next considered the dynamics of neural membrane potentials. Previously, Yu & Ferster (Yu and Ferster, 2010) reported that, in area V1, visual stimulation increases gamma-band correlations between pairs of neural membrane potentials (Figure 7a). Qualitatively similar results were obtained with our model (Figure 7b). Increasing the amplitude of the feed-forward input led to increased correlations between neural membrane potentials (Figure 7c), with strongest coherence observed in the gamma-band range (Figure 7d). This is because more neurons fire spikes on each cycle, leading to stronger oscillations.

**Figure 8.**
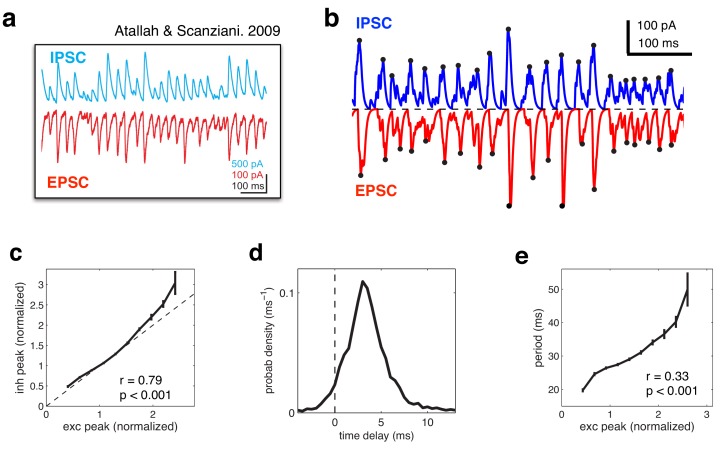
Balanced fluctuations in excitatory and inhibitory currents. (**a**) Data from Atallah & Scanziani (Neuron, 2009), showing inhibitory (blue) and excitatory (red) postsynaptic currents. (**b**) Inhibitory (blue) and excitatory currents (red) in the model, in response to a constant feed-forward input. Black dots indicate detected peaks. (**c**) Amplitude of inhibitory current versus magnitude of excitatory currents on each cycle. (**d**) Distribution of time lags between excitatory and inhibitory peaks. (**e**) Period between excitatory peaks, versus the peak amplitude on the previous oscillation cycle. **DOI:** http://dx.doi.org/10.7554/eLife.13824.009

Several studies have reported a tight balance between inhibition and excitation (Atallah and Scanziani, 2009; Wehr and Zador, 2003; Cafaro and Rieke, 2010). Recently, Atallah et al. (Atallah and Scanziani, 2009) reported that inhibitory and excitatory currents are precisely balanced on individual cycles of an ongoing gamma oscillation (Figure 8a). In our model, efficient coding is achieved by maintaining such a tight balance between inhibitory and excitatory reconstructions. Thus, inhibitory and excitatory currents closely track each other (Figure 8b), with a high correlation between the amplitude of inhibitory and excitatory currents on each cycle (Figure 8c). In common with Atallah et al.’s data, inhibition lags behind excitation by a few milliseconds (Figure 8d). Fluctuations in the amplitude of inhibitory and excitatory currents instantaneously modulate the oscillation frequency, with a significant correlation observed between the peak amplitude on a given oscillation cycle and the period of the following cycle (Figure 8e).

### Gamma oscillations and behavioural performance

**Figure 9.**
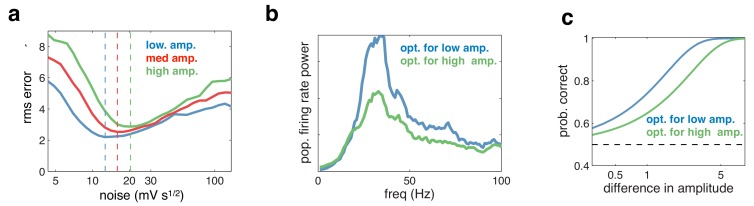
Effect of varying the input amplitude. (**a**) Root-mean-square (rms) reconstruction error versus injected noise amplitude with three different input amplitudes. The optimal noise level in each condition is denoted by vertical dashed lines. (**b**) Power spectrum of population firing rate, in response to a low amplitude input. The blue curve corresponds to when the network is optimised for the low amplitude input, the green curve for when it is optimised for higher amplitude input. (**c**) Fractional performance in discriminating between two 100 ms input segments, equally spaced around the low amplitude input. Performance is best in the condition with strongest oscillations. **DOI:** http://dx.doi.org/10.7554/eLife.13824.010

In general, the optimal network parameters depend on the properties of the feed-forward sensory input. For example, the higher the input amplitude, the more noise is required to achieve the optimal level of network synchrony (Figure 9a). While the network achieves reasonable coding accuracy for a large range of different inputs, adaptive tuning of the dynamics (for example, changing the noise level) can be beneficial for a more limited input range. This would affect the level of population synchrony and thus introduce a correlation between performance and the strength of Gamma oscillations.

For example, if the task is to detect weak (low amplitude) inputs, performance would be higher if top down modulations (such as attention) reduced the level of noise, and thus increased the degree of population synchrony (Figure 9b) without significantly changing firing rates. We can thus expect higher detection performance to correlate with a stronger level of Gamma oscillations (Figure 9c). This could account for certain attention-dependent increases in gamma-power and its correlation with behavioural performance (see **Discussion**).

Note that a similar correlation between behavioural performance and Gamma power could arise from purely bottom up effects. In the presence of input noise causing trial-by-trial changes in input strength, trials with stronger input amplitude would result in more detection but also exhibit more Gamma oscillations. In that case, however, the increase in Gamma power would be associated with a commensurate increase in population firing rate.

### Sensitivity to network paramaters

**Figure 10.**
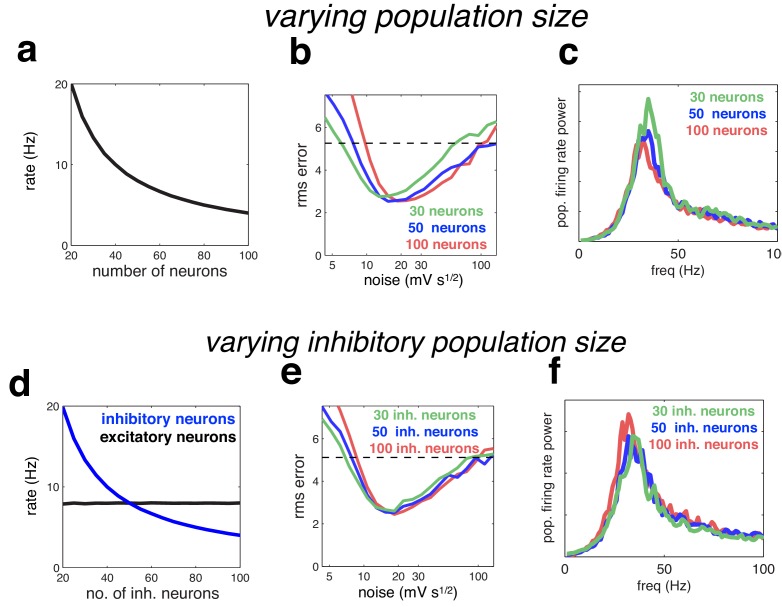
Effect of varying population size. (**a**) With recurrent and feed-forward weights held constant, neural firing rates are inversely proportional to population size. (**b**) Root-mean-squared (rms) reconstruction error versus added membrane noise, for three population sizes. A horizontal line denotes the performance of a Poisson spiking network with matching population firing rate. (**c**) Power spectrum of population of population firing rate, for each network size (in each case, the noise was set to its optimal value). (**d**–**e**) Same as panels **a**–**c**, but where we only varied the size of the inhibitory population. **DOI:** http://dx.doi.org/10.7554/eLife.13824.011

We investigated the degree to which our results depended on the network size, and the ratio of inhibitory to excitatory neurons. With readout weights held constant, neural firing rates in our model are inversely proportional to the network size (such that the population firing rate stays constant; Figure 10a). The optimal coding performance was practically unaltered by increasing or decreasing the population size by a factor of two, although there was a small trend for the optimal noise magnitude to increase with population size (Figure 10b). The oscillatory dynamics were unaltered by varying the network size (Figure 10c). Varying the number of inhibitory neurons (while keeping the number of excitatory neurons constant), had a similar effect on inhibitory firing rates (Figure 10d), with little effect on coding performance or oscillatory dynamics (Figure 10e–f).

**Figure 11.**
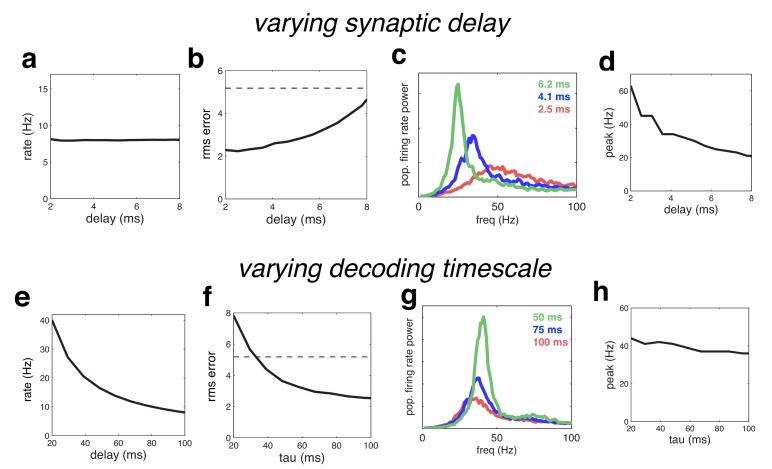
Effect of varying the network time constants. (**a**) Neural firing rates versus the synaptic delay. (**b**) Root-mean-squared (rms) reconstruction error versus the synaptic delay. A horizontal line denotes the performance of a Poisson spiking network with matching population firing rate. (**c**) Power spectrum of population firing rate, for three different settings of the synaptic delay. (**d**) Peak oscillation frequency, versus synaptic delay. (**e**–**h**) Same as panels **a**–**d**, but where we varied the read-out time constant (which determines the effective membrane time constant). **DOI:** http://dx.doi.org/10.7554/eLife.13824.012

We next investigated the effect of varying the time constants of the network. We first rescaled all the time-constants for the synaptic waveform. For plotting purposes, the effective ‘synaptic delay’ was defined as the time for the cumulative input due to a single synaptic event to attain half its maximum value. Increasing the synaptic delay meant that more neurons fired on each oscillation cycle before receiving recurrent inhibition, and thus resulted in decreased coding performance (Figure 11 b), and larger, lower frequency oscillations (Figure 11c).

Because there are only two time constants in the model network, when feed-forward and recurrent weights are rescaled appropriately, decreasing the read-out time, τ, is equivalent to increasing the synaptic delay. However, when recurrent weights are held constant, decreasing τ increases firing rates (which are inversely proportional to τ) in contrast to varying the synaptic delay, which has no effect on firing rates (compare Figure 11 a and e). Decreasing τ also increases the oscillation magnitude with a resulting decrease in coding quality (Figure 11f–g). However, unlike varying the synaptic delay, τ has a relatively weak effect on the oscillation frequency (Figure 11h). Intuitively, this is because varying τ causes two changes in network dynamics, which have opposite effects on the oscillation frequency. On the one hand, decreasing τ results in a faster integration time, speeding up the network dynamics (and thus, increasing the oscillation frequency). On the other hand, decreasing τ increases the oscillation magnitude, leading to stronger inhibition on each oscillation cycle, and thus decreasing the oscillation frequency.

## Discussion

We present a novel hypothesis for the role of neural oscillations, as a consequence of efficient coding in recurrent neural networks with noise and synaptic delays. In order to efficiently code incoming information, neural networks must trade-off two competing demands. On the one hand, to ensure that each spike conveys new information, neurons should actively desynchronise their spike trains. On the other hand, to do this optimally, neural membrane potentials should encode shared global information about what has already been coded by the network, which will tend to synchronise neural activity.

In a network of LIF neurons with dynamics and connectivity tuned for efficient coding, we found that this trade-off is best met when neural spike trains are entrained to a global oscillatory rhythm (implying a consistent representation of the prediction error), but where few neurons fire spikes on each cycle (implying high efficiency). This also corresponds to the regime in which inhibition and excitation are most tightly balanced. Our results provide a functional explanation for why cortical networks operate in a regime in which: (i) global oscillations in population firing rates occur alongside individual neurons with low, irregular, firing rates (Brunel and Hakim, 1999) (ii) there is a tight balance between excitation and inhibition (Atallah and Scanziani, 2009; Wehr and Zador, 2003; Cafaro and Rieke, 2010).

For simplicity, we considered a homogeneous network with one-dimensional feed-forward input. However, the results presented here can be generalised to networks with heterogenous connection, as well as networks that encode high-dimensional dynamical variables.

### Relation to balanced network models

Previously, Brunel & colleagues derived the conditions under which a recurrent network of integrate-and-fire neurons with sparse irregular firing rates exhibits fast global oscillations (Brunel and Hakim, 1999; Renart et al., 2010; Brunel and Wang, 2003; Mazzoni et al., 2008). This behaviour is qualitatively similar to the network dynamics observed in our model. However, these previous models differ in several ways from the model presented here. For example, they assume sparse connections (and/or weak connectivity), in which the probability of connections (and/or connection strengths) scales inversely with the number of neurons. In contrast, the connections in our network are non-sparse and finite. Thus, our network achieves a tighter balance between inhibitory and excitatory currents, and smaller fluctuations in membrane potentials (they scale as 1/N in the absence of delays, rather than $1/\sqrt{N}$).

Previous models of balanced neural networks typically consider medium to large neural populations, for which finite size effects are limited. In contrast, the recurrent network proposed here is particularly relevant to networks with a relatively small number of neurons (i.e. hundreds, rather than thousands, of neurons per input dimension). This is because in cases where a very large number of neurons encode a low-dimensional input, fluctuations in firing rate due to Poisson noise can be averaged out, and thus are not a limiting factor for coding accuracy. Note that the population size at which our proposed recurrent coding scheme ceases to be advantageous depends on several factors, including the input dimensionality and average neural firing rates.

The most important distinction between our work and previous mean-field models lies in the way the network is constructed. In our work, the network connectivity and dynamics are derived from functional principles, in order to minimise a specific loss function (i.e. the squared difference between the neural reconstruction and input signal). This ‘top-down’ modelling approach allows us to directly ask questions about the network dynamics that subserve optimal efficient coding. For example, balanced inhibition and excitation are not imposed in our model, but rather, required for efficient coding. Further, while previous models showed mechanistically how fast oscillations can emerge in a network with slow irregular firing rates (Brunel and Hakim, 1999), our work goes further, showing that these dynamics are in fact required for optimal efficient coding.

**Figure 12.**
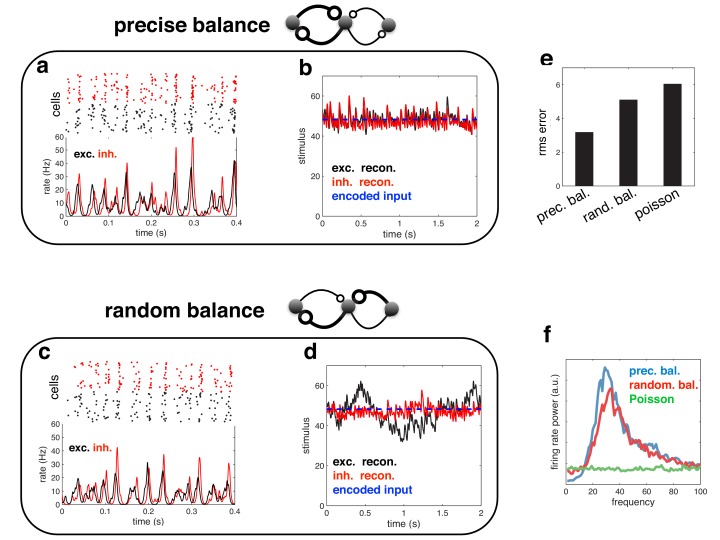
Spiking response (**a**), and neural reconstruction (**b**) of a network with heterogenous read-out weights (and connection strengths). (**c**–**d**) Similar to panels **a**–**b**, except with randomized recurrent connections. (**e**) Root-mean-squared reconstruction error for each model variant, including a population of Poisson units with matching firing rates. (**f**) Power spectrum of population firing rate for each model variant. **DOI:** http://dx.doi.org/10.7554/eLife.13824.013

Finally, it is important to realise that, while efficient coding in a recurrent network leads to global oscillations, the reverse is not true: just because a network oscillates, does not mean that it is performing efficiently. To demonstrate this point, we repeated our simulations in a network with heterogeneous read-out weights (Figure 12a–b). Both the coding performance and spiking dynamics of this network were indistinguishable from the homogeneous network described in the main text. In contrast, when we randomised the recurrent connection strengths (Figure 12c–d; see Materials and methods), the coding performance of the network was greatly reduced (Figure 12e), despite the fact that the network dynamics and firing rate power spectrum were virtually unchanged (Figure 12f).

Indeed, the coherence between excitatory and inhibitory current oscillations (i.e. the level of balance) is a much more reliable signature of efficient coding than global population synchrony. While population synchrony can occur in globally balanced network as well, only networks with an intracellular detailed balance between excitatory and inhibitory currents achieve high coding performance.

### Relation to previous efficient coding models

Previous work on efficient coding has mostly concentrated on using information theory to ask what information ‘should’ be represented by sensory systems (Simoncelli and Olshausen, 2001). Recently, however, researchers have begun to ask, mechanistically, how neural networks should be setup in order to operate as efficiently as possible (Boerlin et al., 2013; Boerlin and Denève, 2011; Deneve, 2008). This approach can provide certain insights not obtainable from information theory alone.

For example, according to information theory, in the low-noise limit, the most efficient spiking representation will be one in which the spike trains of different neurons are statistically independent, and thus, there are no oscillations. In practice however, neural networks must operate in the face of biological constraints, such as synaptic delays. Considering these constraints changes our conclusions. Specifically, contrary to what one might expect from a purely information theoretical analysis, we find that oscillations emerge (even in a regime with low input-noise) as a consequence of neurons performing as efficiently as possible given synaptic delays, and should not be removed at all cost.

### Relation to previous predictive spiking models

Previously, Deneve, Machens and colleagues described how a population of spiking neurons can efficiently encode a dynamic input variable (Boerlin et al., 2013; Boerlin and Denève, 2011; Deneve, 2008; Bourdoukan et al., 2013). In this work, we showed that a recurrent population of integrate-and-fire neurons with dynamics and connectivity tuned for efficient coding maintains a balance between excitation and inhibition, exhibits Poisson-like spike statistics (Boerlin et al., 2013), and is highly robust against perturbations such as neuronal cell death. However, we did not previously demonstrate a relation between efficient coding and neural oscillations. The main reason for this is that we always considered an idealised network, with instantaneous synapses. In this idealised network, only one cell fires at a time. As a result, oscillations are generally extremely weak and fast (with frequency equal to the population firing rate), and thus, completely washed out in a large network with added noise and/or heterogenous read-out weights. In contrast, in a network with finite synaptic delays, more than one neuron may fire per oscillation cycle, before the arrival of inhibition. As a result, oscillations are generally much stronger, even with significant added noise and/or heterogenous read-out weights.

### Attentional modulation

Directing attention to a particular stimulus feature/location has been shown to increase the gamma-band synchronisation of visual neurons that respond to the attended feature/location (Fries et al., 2001). Figure 9 illustrates how such an effect could come about. Here, we show that attentional modulations that increase the strength of gamma-band oscillations will serve to increase perceptual discrimination of low contrast stimuli. Such attentional modulation could be achieved in a number of different ways, for example, by decreasing noise fluctuations, or modulating the effective gain of feed-forward or recurrent connections (Reynolds and Heeger, 2009; Mitchell et al., 2007).

In general, the way in which attention should modulate the network dynamics will depend on the stimulus statistics and task-setup. Future work, that considered higher dimensional sensory inputs (as well as competing ‘distractor’ stimuli), could allow us to investigate this question further.

### The benefits of noise

An interesting aspect of our work is that it suggests various sources of noise (e.g. such as synaptic failure), often thought of as a ‘problem’ for neural coding, may in fact help neural networks achieve higher coding performance than would otherwise be possible.

With low noise, neural membrane potentials in our model are highly correlated (Figure 4a), and inhibition is not able to prevent multiple neurons firing together. To avoid this, we needed to add noise to neural membrane potentials (while simultaneously increasing spiking thresholds). With the right level of noise, fewer neurons fired on each oscillation cycle, resulting in increased coding performance. Too much noise, however, led to inconsistent information being encoded by different neurons, decreasing coding performance (Figure 4c). This phenomena, where noise fluctuations increase the signal processing performance of a system, is often referred to as ‘stochastic resonance’ (Benzi et al., 1981; Faisal et al., 2008), and has been observed in multiple sensory systems, including cat visual neurons (Longtin et al., 1991; Levin and Miller, 1996). Previously however, stochastic resonance has usually been seen as a method to amplify sub-threshold sensory signals that would not normally drive neurons to spike. Here, in contrast, noise desynchronises neurons that receive similar recurrent inputs, increasing the coding efficiency of the population.

### Biological limitations

While it is interesting that, starting from a pure top-down coding rule, one can arrive at a network of recurrently coupled effective integrate-and-fire (IF) neurons, we emphasize that this derived network is still far from being ‘biologically realistic. In the current paper, we address a fundamental limitation of the idealized model that emerges from the derivation, where neural feedback is both noiseless and instantaneous. We show how efficient neural coding can be performed in a network where feedback is noisy and delayed, with resulting oscillatory dynamics that resemble what is observed experimentally.

Nonetheless, significant challenges remain in order to draw a closer connection between the top-down neural model and biology. For example, neurons in our model have voltage-driven synapses, while real synapses are better approximated by conductance based models. In another study, we showed how to adapt the framework to more realistic Hodgkin Huxley neurons that included synapses with finite rise time, but no delays (Schwemmer et al., 2015). Further, the slow time constant (100 ms), required in our simulations to achieve high coding performance, contrasts with the relatively fast (∼10–20 ms) membrane time constants observed experimentally. It will be important in the future to investigate how extensions to the model (such as higher-dimensional inputs and/or alternative implementations with sparse recurrent connectivity) can help recover coding performance given fast, biologically realistic, membrane time constants.

### Alternative functional roles for oscillations

Neural oscillations have been hypothesised to fulfill a number of different functional roles, including feature binding (Singer, 1999), gating communication between different neural assemblies (Fries, 2005; Womelsdorf et al., 2007; Akam and Kullmann, 2010), encoding feed-forward and feed-back prediction errors (Arnal et al., 2011; Arnal and Giraud, 2012; Bastos et al., 2012) and facilitating ‘phase codes’ in which information is communicated via the timing of spikes relative to the ongoing oscillation cycle (Buzsáki and Chrobak, 1995).

Many of these theories propose new ways in which oscillations encode incoming sensory information. In contrast, in our work network oscillations do not directly code for anything, but rather, are predicted as a consequence of efficient rate coding, an idea whose origins go back more than 50 years (Barlow, 1961).

## Materials and methods

### Efficient spiking network

We consider a dynamical variable that evolves in time according to:

(1) $$\tau \,\frac{d\,x\,\left(t\right)}{d\,t}=-x\,\left(t\right)+c\,\left(t\right),$$

where c⁢(t) is a time-varying external input or command variable, and τ is a fixed time constant. Our goal is to build a network of N neurons that take c⁢(t) as input, and reproduce the trajectory of x⁢(t). Specifically, we want to be able to read an estimate $\hat{x}\,\left(t\right)\approx x\,\left(t\right)$ of the dynamical variable from the network’s spike trains $o\,\left(t\right)=\left(o_{1}\,\left(t\right),o_{2}\,\left(t\right),\ldots ,o_{N}\,\left(t\right)\right)$. These output spike trains are given by $o_{i}\,\left(t\right)=\sum_{k}\delta \,\left(t-t_{i}^{k}\right)$, where $t_{i}^{k}$ is the time of the $k^{t\,h}$ spike in neuron i.

We first assume that the estimate, $\hat{x}\,\left(t\right)$, can be read out by a weighted leaky integration of spike trains:

(2) $$\tau \,\frac{d\,\hat{x}\,\left(t\right)}{d\,t}=-\hat{x}\,\left(t\right)+\sum_{i}w_{i}\,o_{i}\,\left(t\right),$$

where $w_{i}$ is a constant read-out weight associated with the $i^{t\,h}$ neuron. For simplicity, we set the readout time-constant, τ, equal to the timescale of the input, x.

We next assume that the network minimises the distance between x⁢(t) and $\hat{x}\,\left(t\right)$ by optimising over spike times $t_{i}^{k}$. The network minimises the loss function,

(3) $$E\,\left(t\right)={\left(x\,\left(t\right)-\hat{x}\,\left(t\right)\right)}^{2}+\alpha \,\sum_{i}r\,\left(t\right)+\beta \,\sum_{i}r_{i}\,{\left(t\right)}^{2}.$$

The first term in the loss function is the squared distance between the x⁢(t) and $\hat{x}\,\left(t\right)$. The second term and third term represent L1 and L2 penalties on the firing rate, respectively. α and β are constants that determine the size of the penalty. The time varying firing rate of the $i^{t\,h}$ neuron is defined to by the differential equation:

(4) $$\tau \,\frac{d\,r_{i}}{d\,t}=-r_{i}+o_{i}\,\left(t\right).$$

A neuron fires a spike at time t if it can reduce the instantaneous error E⁢(t) (i.e. when E(t|neuron  i  spikes) < E(t|neuron  i  doesn't  spike)). This results in a spiking rule:

(5) $$V_{i}\left(t\right)\;>\;T_{i}$$

where,

(6) $$\begin{array}{l} V_{i}\left(t\right)=w_{i}\left(x\left(t\right)-\hat{x}\left(t\right)\right)-\beta r_{i} \end{array}$$

(7) $$\begin{array}{l} T_{i}=\frac{1}{2}\left(w_{i}^{2}+\alpha +\beta \right). \end{array}$$

Since $V_{i}\,\left(t\right)$ is a time-varying variable, whereas $T_{i}$ is a constant, we identify the former with the $i^{t\,h}$ neuron’s membrane potential $V_{i}\,\left(t\right)$, and the latter with its firing threshold $T_{i}$.

To obtain the network dynamics, we take the derivative of each neuron’s membrane potential to obtain:

(8) $$\tau \,\frac{d\,V_{i}\,\left(t\right)}{d\,t}=-V_{i}\,\left(t\right)+w_{i}\,c\,\left(t\right)-w_{i}\,\sum_{k}w_{k}\,o_{k}\,\left(t\right)-\beta \,o_{i}\,\left(t\right)+\sigma \,\nu_{i}\,\left(t\right).$$

where $\nu_{i}\,\left(t\right)$ corresponds to a white ‘background noise’, with unit variance (added for biological realism). Thus, the resultant dynamics are equivalent to a recurrent network of leaky integrate-and-fire (LIF) neurons, with leak, -V⁢(t), feed-forward input, $w_{i}\,c\,\left(t\right)$, recurrent input, $-w_{i}\,\sum_{k}w_{k}\,o_{k}\,\left(t\right)$, and self-inhibition (or reset), $-\beta \,o_{i}\,\left(t\right)$. Note that in the case considered here, where the decoding timescale is equal to the membraine time constant, the ‘leak term’, $V_{i}$, emerges directly from the derivation.

### Balanced network of inhibitory and excitatory neurons

To construct a network that respects the Dale’s law, we introduce a population of inhibitory neurons, that tracks the estimate encoded by the excitatory neurons, and provides recurrent feedback. For simplicity, we consider a network in which all read-out weights are positive. In our framework, this results in a particularly simple network architecture, in which a single population of excitatory neurons is recurrently connected to a population of inhibitory neurons (Figure 3a). For further discussion of different network architectures, see (Boerlin et al., 2013).

We first introduce a population of inhibitory neurons, that receive input from excitatory cells. The objective of the inhibitory population is to minimise the squared distance between excitatory and inhibitory neural reconstructions (${\hat{x}}_{E}$, and ${\hat{x}}_{I}$, respectively), by optimising over spike times $t_{i}^{k}$. Thus, an inhibitory neuron spikes when it can reduce the loss function:

(9) $$E\,\left(t\right)={\left({\hat{x}}_{I}\,\left(t\right)-{\hat{x}}_{E}\,\left(t\right)\right)}^{2}+\alpha_{I}\,\sum_{i}r_{i}^{I}\,\left(t\right)+\beta_{I}\,\sum_{i}r_{i}^{I}\,{\left(t\right)}^{2}.$$

Following the same prescription as before, we obtain the following dynamics for the inhibitory neurons:

(10) $$\begin{array}{ll} \tau \frac{dV_{i}^{I}}{dt}= & -V_{i}^{I}\left(t\right)+w_{i}^{I}\sum_{k}w_{k}^{E}o_{k}^{E}\left(t\right)\\ & -w_{i}^{I}\sum_{k}w_{k}^{I}o_{k}^{I}\left(t\right)-\beta_{I}o_{k}^{I}+\sigma \nu_{i}^{I}\left(t\right). \end{array}$$

Thus, inhibitory neurons receive input from excitatory neurons (second term), and recurrent inhibition from other inhibitory neurons (third term).

Now, as the inhibitory reconstruction tracks the excitatory reconstruction, we can make the simplifying assumption of replacing ${\hat{x}}_{E}$ with ${\hat{x}}_{I}$, in our earlier expression for the excitatory membrane potential (equation 6), giving:

(11) $$V_{i}^{E}\,\left(t\right)=w_{i}^{E}\,\left(x\,\left(t\right)-{\hat{x}}_{I}\,\left(t\right)\right)-\beta_{E}\,r_{i}^{E}$$

Taking the derivative of this expression as before, we obtain the following dynamics for the excitatory neurons:

(12) $$\begin{array}{ll} \tau \frac{dV_{i}^{E}}{dt}= & -V_{i}^{E}\left(t\right)+w_{i}^{E}c\left(t\right)\\ & -w_{i}^{E}\sum_{k}w_{k}^{I}o_{k}^{I}\left(t\right)-\beta_{E}o_{k}^{E}+\sigma \nu_{i}^{E}\left(t\right). \end{array}$$

Thus, excitatory neurons receive excitatory feed-forward input (second term) and recurrent inhibitory input (third term).

### Synaptic dynamics

To account for transmission delays and continuous synaptic dynamics we assume that each spike generates a continuous current input to other neurons, with dynamics described by the synaptic waveform, $h\,\left(t-t_{i}^{l}\right)$. The shape of this waveform is given by:

(13) $$h\left(t\right)=\left\{ \begin{array}{ll} \frac{1}{\tau_{d}-\tau_{r}}\left[\exp\left(\frac{-\left(t-\tau_{tr}\right)}{\tau_{d}}\right)-\exp\left(\frac{-\left(t-\tau_{tr}\right)}{\tau_{r}}\right)\right] & \text{ if }t\;>\;\tau_{tr}\\ 0 & \text{ if }t\;>\;\tau_{tr} \end{array}\right.$$

where $\tau_{r}$ is the synaptic rise time, $\tau_{d}$ is the decay time and $\tau_{t\,r}$ is the transmission delay. The normalisation constant, $\tau_{d}-\tau_{r}$, ensures that $\int_{\tau_{t\,r}}^{\infty }h\,\left(t\right)\,d\,t=1$. This profile is plotted in Figure 3b, with $\tau_{r}=1\,m\,s$, $\tau_{d}=3\,m\,s$ and $\tau_{t\,r}=1\,m\,s$.

To incorporate continuous synaptic currents into the model, we alter equations 10 and 12, by replacing each of the recurrent spiking inputs ($o_{k}\,\left(t\right)$) by the convolution of the spiking input and current waveform ($h\left(t\right)⋆o_{k}\left(t\right)\leftarrow o_{k}\left(t\right)$).

### Simulation parameters

For the simulations shown in Figure 2, we considered a toy network of 3 neurons with equal read-out weights, $w_{i}=1$. The L1 spike cost was α=0 and the L2 spike cost was set to β=0.04. The read-out time constant was set to τ=0.1⁢s. The magnitude of injected membrane potential noise was set to σ=0.02. In each case, network dynamics were computed from equation 8.

For the simulations shown in Figures 3–12, we considered a larger network of 50 excitatory neurons, and 50 inhibitory neurons. All neurons had equal read-out weights, equal to $\gamma_{0}=1.2\,{\mathrm{mV}}^{1/2}$. The L1 spike cost was set to 0. The L2 spike cost was set to β=8.5 mV. If we assume a spike threshold of −55 mV, this corresponds to a resting potential of −60 mV ($V_{r\,e\,s\,t}=V_{t\,h\,r\,e\,s\,h}-\frac{1}{2}\,\left(L_{1}+L_{2}+\gamma_{0}^{2}\right)$), a reset of −65 mV ($V_{r\,e\,s\,e\,t}=V_{t\,h\,r\,e\,s\,h}-L_{2}-\gamma_{0}^{2}$), and post-synaptic potentials of 1.45 mV ($V_{P\,S\,P}=\gamma_{0}^{2}$; se*e (Boerlin et al., 2013) *for details of scaling to biological parameters).

For Figures 3, 6–8, 10 and 12 the magnitude of injected membrane potential noise was set to its ‘optimal value’ σ=17 mV $s^{1/2}$. For Figure 5 the membrane potential noise was set lower, at σ=8 mV $s^{1/2}$.

Network dynamics were computed from equations 10 and 12 (with the exception that recurrent inputs were convolved with the synaptic current waveform, h⁢(t), described in equation 13).

### Algorithm

Simulations were run using a Euler method, with discrete time steps of 0.5 ms. For the ‘ideal’ network (i.e. with instantaneous synapses) only one neuron (with highest membrane potential) was allowed to fire a spike within each bin. Changing the temporal discretisation did not qualitatively alter our results.

### Stimulus details

In Figure 2b, the encoded variable, x, was obtained by low-pass filtering white noise with a first-order Butterworth filter, with cut-off frequency of 4 Hz (the low-pass Butterworth filter, with transfer function proportional to $1/\left(1+{\left(\omega /\omega_{c}\right)}^{2\,n}\right)$, where n is the filter order, and $\omega_{c}$ is the cut-off frequency, was implemented using Matlab’s ‘butter’ command).

After filtering, x, is rescaled to have a mean of 3, and standard deviation of 1. In Figure 2c the encoded variable is constant, x=4. In Figure 3, the encoded variable is obtained by low-pass filtering a white-noise input in the same way as for Figure 2b, this time with a cut-off frequency of 2 Hz. After filtering, x, is rescaled to have non-zero mean of 50, and standard deviation of 10. In Figures 4–5, 7–8 and 10–12 the encoded variable was held constant at x=50. In Figures 6 and 9, the ’low’, ’medium’ and ’high’ amplitude inputs are x= 35, 50, and 65, respectively.

### Poisson model

In Figure 3d–e, we compare the efficient coding model to a rate model, in which neural firing rates vary as a function of the feed-forward input, c⁢(t). Firing rates for the Poisson model were equal to the mean firing rate in the recurrent model (when excitatory readout weights are all equal, firing rates are proportional to the feed-forward input, c⁢(t)). Spiking responses were obtained by drawing from a Poisson distribution.

### Varying the noise

For the simulations shown in Figure 4, we varied the injected membrane potential noise. In general, varying the noise amplitude changes neural firing rates, leading to systematic estimation biases. To compensate for this, we adjusted the L2 spike cost for the inhibitory and excitatory neurons, so as to maintain zero estimation bias (or equivalently, to keep firing rates constant). For each noise level, we ran an initial simulation, modifying excitatory and inhibitory costs, $\beta_{E}$ and $\beta_{I}$, in real time (via a stochastic gradient descent algorithm), until both the excitatory and inhibitory estimation biases converged to zero.

To generate Figure 5a–b we varied the synaptic reliability, by altering the probability that a presynaptic spike produces a change in the postsynaptic membrane potential. To keep the total synaptic input to each neuron constant, we divided the recurrent connection strengths by the probability of synaptic failure.

To generate Figure 5c–d we chose a fraction of inhibitory and excitatory neurons. The selected neurons fired spikes random Poisson spike trains. Their firing rates remained unchanged.

### Population firing rates

To plot the population firing rate (Figure 6a and b), we low-pass filtered neural spike trains using a first order Butterworth filter (with cut-off frequency of 5.5 Hz, and 66 Hz, for panels b and d, respectively), before averaging over neurons.

### Spectral analysis

The spectrogram of the population firing rate, shown in Figure 6a (lower panel), was computed using a short-time Fourier-transform, with a Hamming time window of 60 ms (Matlab’s ‘spectrogram function’). Finally, the instantaneous power spectrum was low-pass filtered with a first-order Butterworth filter, with cut-off frequency 3 Hz. The power spectrum of the population firing rate and neural membrane potentials (Figures 4h, 5b and d, 6c) was computed using the multi-taper method (using Matlab’s ‘pmtm’ function), with bandwidth chosen empirically to achieve a **s**pectrum that varied smoothly with frequency.

### Excitatory and inhibitory currents

To plot the currents shown in Figure 8b, we divided the total excitatory and inhibitory input to each cell by a presumed membrane resistance of $R_{m}=5\,\text{M}\,\Omega$ (changing this value rescales the y-axis). We then defined peaks in excitatory and inhibitory currents as local maxima in the currents, separated by a drop an 80% drop in the current magnitude. Further, we only included peaks in inhibitory and excitatory currents that occurred within 15 ms of each other.

### Discrimination threshold

For the simulation shown in Figure 9, we considered the performance of the network in discriminating between two 0.1 s long stimulus segments, with equally spaced around the ‘low’ amplitude input (x=48). From signal detection theory, a subject’s probability of selecting between two stimuli is given by: $P_{c\,o\,r\,r\,e\,c\,t}\,\left(x_{1},x_{2}\right)=\frac{1}{2}\,\mathrm{erfc}\,\left(\frac{1}{2}\,D\,\left(x_{1},x_{2}\right)\right)$, where erfc⁢(x) is the cumulative error function, and $D\,\left(x_{1},x_{2}\right)$ is the normalized distance (or d-prime) between the distribution of estimates: $D\,\left(x_{1},x_{2}\right)=\frac{\mu \,\left(x_{2}\right)-\mu \,\left(x_{1}\right)}{\sqrt{\frac{1}{2}\,\sigma^{2}\,\left(s_{1}\right)+\sigma^{2}\,\left(s_{2}\right)}}$. These quantities can be directly computed from the network output.

### ‘Random’ versus ‘precisely’ balanced network

The network used to generate Figure 12a–b was the same as before, with the exception that the readout weights varied (and thus, the connection strengths) for each neuron. Readout weights were sampled from a gamma distribution, with mean $1.2\,{\mathrm{mV}}^{1/2}$, and standard deviation $0.5\,{\mathrm{mV}}^{1/2}$. For the optimal ‘precisely balanced’ network (Figure 12a–b), the reciprocal connection strengths between each inhibitory and excitatory neuron are equal in magnitude. For the ‘randomly’ balanced network (Figure 12c–d), we disrupted this symmetry by randomly permuting the input to each excitatory/inhibitory neuron (while ensuring that the summed input to each neuron remained unchanged). Recurrent inhibitory connections were left unchanged.
