# Supplementary material for: Neural oscillations as a signature of efficient coding in the presence of synaptic delays
Source: eLife. 2016 Jul 7;5:e13824. doi: 10.7554/eLife.13824 (PMC4959845; doi:10.7554/eLife.13824)
Supplement: Related texts

# Article commentary: Decision letter

**DOI:** 10.7554/eLife.13824.014

**Affiliations:** University College London, United Kingdom

In the interests of transparency, eLife includes the editorial decision letter and accompanying author responses. A lightly edited version of the letter sent to the authors after peer review is shown, indicating the most substantive concerns; minor comments are not usually included.

Thank you for submitting your work entitled "Neural oscillations as a signature of efficient coding in the presence of synaptic delays" for consideration by *eLife*. Your article has been favorably evaluated by Timothy Behrens as the Senior editor and three reviewers, one of whom is a member of our Board of Reviewing Editors.

The reviewers have discussed the reviews with one another and the Reviewing Editor has drafted this decision to help you prepare a revised submission.

Summary:

The authors have previously shown that a an optimal error correcting code requires a balanced excitatory (E) and inhibitory (I) network, where a spike occurs only to reduce the error between a network estimate and the true stimulus. In that previous work, feedback was instantaneous. Here the authors show that if feedback is delayed (as it must be in realistic networks), oscillations develop. However, excessive synchronous network oscillations degrades coding. The central result of the manuscript is that noise mitigates some of the deleterious aspects of network-wide oscillatory synchrony on neural coding.

Essential revisions:

1) Some features of the result will naturally depend on the readout time constant τ. The only place I see its value mentioned is in connection with Figure 2 (subsection "Simulation parameters", first paragraph). There it says that τ was 100 ms. Was this value also used in the later simulations? It seems rather a large value to want to associate with a membrane time constant, so I think it would be useful if the authors said something about how this filtering might be implemented biologically. And what would the results look like if τ had a value more like a typical membrane time constant?

2) How sensitive are the results to the parameters of the network? In particular, how do they scale with network size, and with the ratio of excitatory to inhibitory neurons? In particular, when the network is scaled up and the ratio of excitatory to inhibitory neurons is set to a more realistic value, like 4, what happens to the following:

A) Does the optimal noise (Figure 4c) stay at 15 mV? And does the ratio of the optimal RMS error to the Poisson RMS error stay the same?

B) Does the optimal failure probability stay at about 0.5? And does the ratio of the optimal RMS error to the Poisson RMS error (which should be shown in that figure) stay the same?

C) Do the oscillation frequencies stay in the 30-50 Hz range?

D) Do the oscillation frequencies depend most strongly on the delay, or on other network parameters?

3) In the text referring to Figure 3(e), it would be good to explain why the rate in the performance-matched case is so high.

4) The fact that failures improves network performance may be one of the most interesting results in the paper, as it implies that failures are a feature, not a bug. We suggest that the paper would have more impact on the community if you emphasized this point, although we will leave that up to you.

# Reply: Author response

**DOI:** 10.7554/eLife.13824.015

*Essential revisions:*

1) Some features of the result will naturally depend on the readout time constant τ. The only place I see its value mentioned is in connection with Figure 2 (subsection "Simulation parameters", first paragraph). There it says that τ was 100 ms. Was this value also used in the later simulations? It seems rather a large value to want to associate with a membrane time constant, so I think it would be useful if the authors said something about how this filtering might be implemented biologically. And what would the results look like if τ had a value more like a typical membrane time constant?

There are two time constants in our model, the readout time, τ, and the synaptic delay. Thus (with other parameters rescaled accordingly), reducing the readout time constant is equivalent to increasing the delay. We conducted additional simulations (Figure 11) showing that both decreasing τ or increasing the synaptic delay have similar effects on coding performance, which is reduced. These simulations are described in more detail later, in our response to the second comment raised by the reviewers.

To retain coding performance with a smaller read-out time constant requires introducing changes to the ideal network, to prevent multiple neurons firing synchronously, and dampen the resulting excessive oscillations. In the main text we illustrated how encoding performance can be recovered by adding noise (e.g. synaptic failure, additive membrane potential noise or Poisson spiking neurons). We note that coding performance can also be improved by altering other aspects of the network, such as recurrent connectivity. For example, while beyond the scope of the current work, we found that a locally connected network consisting of several overlapping inhibitory sub-populations, each of which encodes its own ‘version’ of the input, can lead to weaker oscillations, improving the performance of the ‘all-to-all’ network presented in our work.

The reviewers raise the fair point that the decoding time constant of 100 ms in the paper is considerably longer than typical membrane time constants. Indeed, the value of 100 ms was used primarily to ensure consistency with our previous theoretical work (Boerlin et al. 2013, PLoS Comp), rather than for explicit biological realism. It is worth noting however, that in contrast to the simple effective integrate-and-fire (IF) model that results from deriving our framework, real neurons exhibit dynamics on multiple timescales, including slow adaptation time scales (Fairhall et al., 2001, Nature), slow inactivation dynamics of intrinsic conductances (Gilboa et al., 2015, J Neurosci), slow integration due to voltage-dependent potassium currents (Storm et al., 1988, Nature) and slower dendritic integration time-scales due to NMDA-synaptic currents (London et al., 2005, Ann. Rev. Neurosci). It is possible that these slower dynamics could account for the slow readout time, that is required by the network in order to achieve a high degree of coding precision.

Finally, while it is interesting to show how, starting from a pure ‘top-down’ coding rule, one can arrive at a network of recurrently coupled effective integrate-and-fire (IF) neurons, we emphasize that this derived network is still far from being 'biologically realistic’. In the current paper, we addressed a major inconsistency between our previous work, where synapses were noiseless and instantaneous, and biology, where synapses are noisy and delayed. Significantly, we believe that the principles that emerge extend beyond the model network, to many other recurrent systems where interacting sub-units perform a global optimization. Nonetheless, we concede that significant challenges remain in order to draw a closer connection between our top-down neural model and biology: not least the introduction of conductance based synapses and/or understanding how the multiple cellular mechanisms may lead to slow decoding time-scales in spite of fast membrane time constants.

We have added a section to the Discussion (“Biological limitations”), with the above arguments.

*2) How sensitive are the results to the parameters of the network? In particular, how do they scale with network size, and with the ratio of excitatory to inhibitory neurons? In particular, when the network is scaled up and the ratio of excitatory to inhibitory neurons is set to a more realistic value, like 4, what happens to the following:*

*A) Does the optimal noise (Figure 4c) stay at 15 mV? And does the ratio of the optimal RMS error to the Poisson RMS error stay the same?*

*B) Does the optimal failure probability stay at about 0.5? And does the ratio of the optimal RMS error to the Poisson RMS error (which should be shown in that figure) stay the same?*

*C) Do the oscillation frequencies stay in the 30-50 Hz range?*

D) Do the oscillation frequencies depend most strongly on the delay, or on other network parameters?

As suggested by the reviewers, we investigated the behavior of the model network with varying: (i) population size, (ii) inhibitory population size only, (iii) synaptic delay, and (iv) the decoding timescale. These results are presented in two new figures (Figure 10–11), described in a new section in the Results (‘Sensitivity to network parameters’).

With all other parameters held constant, increasing the population size results in a lower firing rate for each neuron (such that the summed firing rate of all neurons is constant; Figure 10a). When only the inhibitory population size was altered, then the inhibitory firing rate varied while the excitatory firing rate is constant (Figure 10d).

The coding performance and oscillatory dynamics, on the other hand, remain relatively unchanged when we vary the population size or inhibitory/excitatory ratio. For example, neither the ‘optimal’ noise level nor the oscillation frequency were greatly changed by increasing/decreasing the population size or excitatory/inhibitory ratio by a factor of two (Figure 10b–c and e–f).

Note that, although in our simulations, varying the ratio of excitatory to inhibitory neurons leads to unequal firing rate for the two populations (Figure 10d), this does not have to be the case: one could rescale the inhibitory/excitatory readout weights so that both populations have equal rates. Nonetheless, whatever the manipulation, EI currents should equal the total IE currents, so that balance in the network is preserved.

Finally, we emphasize that our efficient coding model is particularly applicable for small to medium size neural ensembles (with correspondingly low population firing rates), where noise fluctuations resulting from Poisson spiking would otherwise lead to decreased coding performance (see Figure 3d). Thus, we did not find it relevant to scale our model to represent very large networks (i.e. with 1000s of neurons).

In contrast to variations in the population size, varying the synaptic delay had a significant effect on both network dynamics and coding performance. Increasing the synaptic delay resulted in larger and lower frequency oscillations, with a concomitant decrease in coding accuracy (Figure 11A–d).

There are only two time constants in the network: the delay, and the decoding time constant, τ. Therefore, with other parameters (e.g. feed-forward/recurrent weights) scaled appropriately, decreasing τ is equivalent to increasing the synaptic delay. On the other hand, when all other parameters are held constant, decreasing τ serves to increase firing rates (which are inversely proportional to τ), unlike varying the synaptic delay, which has no effect (compare Figures 11a and e).

In common with increasing the length of the delay, decreasing τ also increases the magnitude of network oscillations, while decreasing the coding quality (Figure 11f–g). However, unlike the delay, varying τ had a relatively weak effect on the oscillation frequency (Figure 11g–h). Intuitively, this is because varying τ causes two different changes in network dynamics that push in different directions. On the one hand, decreasing τ results in faster integration time, speeding up the network dynamics (and thus, tending to increase oscillation frequency). On the other hand, decreasing τ increases the oscillation magnitude, leading to stronger inhibition on each oscillation cycle and tending to slow down the oscillations.

3) In the text referring to Figure 3(e), it would be good to explain why the rate in the performance-matched case is so high.

We added a new figure panel (Figure 3d) to show the relation between firing rate and coding performance in each of the model networks. Instead of showing bar plots of performance at a given fixed rate (or conversely, rate required to achieve a given level of performance), we plot the full error/rate curves for the ‘ideal recurrent’ and Poisson models. The non-ideal recurrent network is shown as a black cross on this plot.

In the Poisson network, random fluctuations in firing rate cause the reconstruction to deviate from its true value, and decrease coding performance. These noise fluctuations become less important as the population firing rate increases, with a corresponding decrease in the reconstruction error (that scales as ~1/√F), where F is the population firing rate).

In contrast, in the ideal efficient coding network, noise fluctuations are automatically ‘corrected for’ by the recurrent connection. Thus, the only source of inaccuracy comes from the discreteness of the code (where each spike adds a fixed quantity to the readout), leading to a much smaller reconstruction error (that scales as ‘1/F’).

With the addition of synaptic delays, it is no longer possible to achieve the performance of the ideal network. Nonetheless, by desynchronizing the network with an appropriate level of noise, this problem can be minimized, leading to a reconstruction error significantly smaller than for the Poisson network.

We have added text to the Results (“Efficient coding with synaptic delays”) to clarify these concepts.

*4) The fact that failures improves network performance may be one of the most interesting results in the paper, as it implies that failures are a feature, not a bug. We suggest that the paper would have more impact on the community if you emphasized this point, although we will leave that up to you.*

We thank the reviewer for this suggestion. We also think that this is an interesting point to make. For simplicity, we chose to continue to use additive noise on the membrane potential for the majority of the simulations (we could redo all of them with the failures without changing the results qualitatively). However, we have added text to the Abstract, Introduction (see final paragraph) and Discussion (‘The benefits of noise’) to emphasize how our work suggests that synaptic failures (and noise in general) may be a feature, not a bug.
